# ﻿*Chromisabadhah* (Teleostei, Pomacentridae), a new species of damselfish from mesophotic coral ecosystems of the Maldives

**DOI:** 10.3897/zookeys.1219.126777

**Published:** 2024-11-29

**Authors:** Luiz A. Rocha, Hudson T. Pinheiro, Ahmed Najeeb, Claudia R. Rocha, Bart Shepherd

**Affiliations:** 1 Department of Ichthyology, California Academy of Sciences, San Francisco, CA 94118 USA; 2 Center for Marine Biology, University of São Paulo, São Sebastião, SP 11600, Brazil; 3 Maldives Marine Research Institute, Ministry of Fisheries, Marine Resources and Agriculture, Malé 20025, Maldives; 4 Department of Microbiology, California Academy of Sciences, San Francisco, CA 94118 USA; 5 Steinhart Aquarium, California Academy of Sciences, San Francisco, CA 94118 USA

**Keywords:** *COI*, deep reefs, ichthyology, Indian Ocean, rebreather diving, taxonomy

## Abstract

A new species of *Chromis* (Teleostei, Pomacentridae) is described from four specimens collected between 95 and 110 m depth in mesophotic coral ecosystems in the Maldives, Indian Ocean. *Chromisabadhah***sp. nov.** can be distinguished from all of its congeners by the following combination of characters: dorsal-fin rays XIII, 12–13; anal-fin rays II,11–12; pectoral-fin rays 17–18; tubed lateral-line scales 17; gill rakers 7+17–18 = 24–25; pearly white body with a large black marking covering the anterior two-thirds of the anal fin. The closest DNA barcode sequence (5.1% average uncorrected genetic distance on the mitochondrial *COI* gene), among those available, is *Chromiswoodsi*, a similar mesophotic species known from the coastal western Indian Ocean (Somalia to South Africa). The new species is easily distinguished from *C.woodsi* by having 13 dorsal spines (versus 14 in *C.woodsi*), the absence of a black band on the base of the tail (present in *C.woodsi*), and by the genetic difference.

## ﻿Introduction

The family Pomacentridae (damselfishes and anemonefishes) is one of the largest and most conspicuous families of fish inhabiting tropical shallow coral reefs (Allen 1991). One of the key elements of their evolutionary success in coral reefs is resource partitioning, both at the spatial and trophic axes, with species falling into three main trophic levels: herbivores, zooplanktivores, and omnivores (Frédérich and Parmentier 2016). Despite its recent splitting into three genera (*Azurina* Jordan & McGregor, 1898, *Pycnochromis* Fowler, 1941, and *Chromis* Cuvier, 1814 sensu stricto; Tang et al. 2021), *Chromis* still is the most species-rich genus within Pomacentridae, comprising 78 valid species, all zooplanktivorous, at least 10 of which, including several recently described, are found only below 60 m (Pyle et al. 2008; Arango et al. 2019; Tea et al. 2019; McFarland et al. 2020). They are conspicuous residents of mesophotic coral ecosystems (coral reef habitats occurring at depths from 30–150 m) across all tropical seas, and are often seen in high numbers where they occur. *Chromis* is also the only genus of Pomacentridae that occurs below 80 m and down to at least 180 m depth (McFarland et al. 2020; Shepherd et al. 2020).

Because they are much harder to explore than their shallow-water counterparts, deep reefs are home to many undescribed species (Pyle et al. 2019). Their relative inaccessibility initially led scientists to consider deep reefs as potential refuges for threatened shallow-water species; however, the general consensus now is that species assemblages in mesophotic ecosystems are very different from shallow-water ones, and that deep reefs are also heavily impacted (Rocha et al. 2018; Pinheiro et al. 2023).

Exploration of the planet’s mesophotic ecosystems has been uneven. Hawaii and some locations in the Caribbean, the South Pacific and the Red Sea are relatively well-known, whereas the eastern Pacific, eastern Atlantic and Indian Ocean are largely unexplored (Bongaerts et al. 2019). Aiming to fill this gap through a partnership between the California Academy of Sciences, the Maldives Marine Research Institute, and Rolex, we conducted ichthyological surveys to depths of 150 meters at 21 locations across five atolls in the Maldives, Indian Ocean between January 2022 and October 2023. Several new species of fishes were discovered during these surveys, of which this is the first to be described.

## ﻿Material and methods

Specimens were collected with hand nets and immediately transported to a field laboratory where they were photographed, tissue sampled, and fixed in 10% formalin. Measurements and x-radiographs were made at the California Academy of Sciences after fixation and preservation in 75% ethanol. Counts were made with the aid of a microscope; measurements were made with digital calipers to the nearest 0.01 mm and rounded to one decimal place, following the conventions described in Allen and Randall (2004) and Pyle et al. (2008). Spiniform procurrent caudal-fin rays are those situated anteriorly to segmented procurrent caudal-fin rays, especially visible in radiographs. Vertebral counts include the first vertebra fused to the skull, and the last vertebra fused to the hypural plate; vertebral counts are presented as precaudal + caudal, where the first caudal vertebrae is the anteriormost vertebrae having its haemal spine associated with the anal fin pterygiophore, and the last caudal vertebrae is fused to the hypural plate. Gill-raker counts are presented as upper (epibranchial) + lower (ceratobranchial) rakers on the anterior face of the first arch; the angle raker is included in the second count. Counts and measurements for the holotype and paratypes are presented in Table 1. Measurements in the text are given as percent standard length (**SL**), unless otherwise noted. Data are presented first for the holotype, followed by a range of values for the paratypes, in parenthesis, where variation was noted. The holotype and paratypes were deposited at the California Academy of Sciences (**CAS**) and at the Smithsonian Institution’s National Museum of Natural History (**USNM**) ichthyological collections.

**Table 1.** Counts and measurements for *Chromisabadhah* sp. nov., holotype and paratypes.

	Holotype	Paratypes		
	CAS 248401	CAS 248403	USNM 470765	CAS 248402
TL (mm)	92.6	90.1	87.1	67.4
Standard length (mm)	68.7	66.1	64.5	50.2
**Counts**:				
Dorsal-fin rays	XIII, 12	XIII, 13	XIII, 12	XIII, 12
Anal-fin rays	II, 11	II, 12	II, 11	II, 11
Pectoral-fin rays	18 | 18	18 | 18	18 | 18	17 | 18
Pelvic-fin rays	I, 5	I, 5	I, 5	I, 5
Principal caudal rays	7 + 6	7 + 6	7 + 6	6 + 6
Procurrent segmented caudal rays	3 | 3	3 | 3	3 | 3	3 | 3
Procurrent spiniform caudal rays	3 | 3	3 | 3	3 | 3	3 | 3
Tubed lateral-line scales	17	17	17	17
Posterior mid-lateral pored scales	8	9	9	9
Scales above l.l.	3	3	3	3
Scales below l.l.	9	9	9	9
Circumped. scales	16	16	17	18
Gill rakers	7 + 17	7 + 18	7 + 18	7 + 17
Supraneural bones	3	3	3	3
Vertebrae	26 (11+15)	26 (11+15)	26 (11+15)	26 (11+15)
**Measurements**:				
Body depth	56.3	59.0	55.5	55.0
Body width	21.1	20.2	18.3	19.6
Head length	35.6	35.6	36.0	36.8
Snout length	9.6	8.9	9.3	9.8
Orbit diameter	15.0	14.2	12.6	14.2
Interorbital width	14.3	12.7	13.4	12.2
Caudal-peduncle depth	14.3	15.4	15.4	15.7
Caudal-peduncle length	7.7	7.8	10.3	8.6
Upper jaw length	12.6	11.5	10.5	10.6
Pre-dorsal length	41.9	42.7	42.1	42.8
Spinous dorsal-fin base	49.4	50.9	49.3	46.2
Soft dorsal-fin base	15.6	16.3	15.2	14.5
Dorsal-fin base	65.0	67.2	62.9	46.0
1^st^ dorsal spine	6.7	8.7	10.3	8.5
2^nd^ dorsal spine	13.4	13.2	15.9	14.9
3^rd^ dorsal spine	15.8	16.8	18.0	18.4
4^th^ dorsal spine	17.5	17.7	19.4	21.4
5^th^ dorsal spine	17.1	17.7	18.8	21.0
6^th^ dorsal spine	18.3	17.6	18.6	20.5
Last dorsal spine	12.1	11.8	11.9	14.4
Longest dorsal ray	25.8 (4^th^)	20.8 (4^th^)	21.7 (4^th^)	28.2 (3^rd^)
Preanal length	73.9	72.4	69.1	67.2
1^st^ anal spine	7.5	7.5	7.2	7.1
2^nd^ anal spine	23.5	22.2	21.9	23.3
Longest anal ray	22.2 (1^st^)	19.4 (1^st^)	19.6 (1^st^)	20.2 (1^st^)
Anal-fin base	21.3	24.7	24.2	21.1
Caudal-fin length	33.9	32.1	39.2	34.7
Caudal concavity	15.0	15.4	20.3	16.4
Longest pectoral ray	34.6 (1^st^)	34.5 (1^st^)	31.6 (1^st^)	32.9 (1^st^)
Prepelvic length	41.3	42.4	40.9	43.0
Pelvic-spine length	20.1	20.6	20.6	23.2
1^st^ pelvic soft ray	29.5	30.4	32.6	29.5

In December 2022, four individuals were collected with hand nets by members of our team diving on mixed-gas, closed-circuit rebreathers (Hollis Prism 2). Molecular analysis and PCR amplification of the standard barcode fragment of the mitochondrial cytochrome *c* oxidase subunit I gene (*COI*) were performed following protocols described by Arango et al. (2019) using BOLFishF1/BOLFishR1 primers. Alignments of DNA sequences were done using a standard Geneious global alignment with free end gaps and 65% similarity in the program Geneious Prime 2020.0.3 (Kearse et al. 2012). Our genetic dataset contains sequences of only one mitochondrial DNA marker. Therefore, we did not attempt to perform a phylogenetic reconstruction, and genetic distances are uncorrected. We used all available *ChromisCOI* sequences from GenBank and the Barcode of Life databases for genetic comparisons.

## ﻿Results

### ﻿ Chromis abadhah sp. nov.

Taxon classificationAnimaliaPerciformesPomacentridae

5B39CF02-1679-5B64-88B6-6A21768118CA

https://zoobank.org/885AA4D1-AFF6-4CBF-91B7-EBBA49760C18

**Figure 1.**
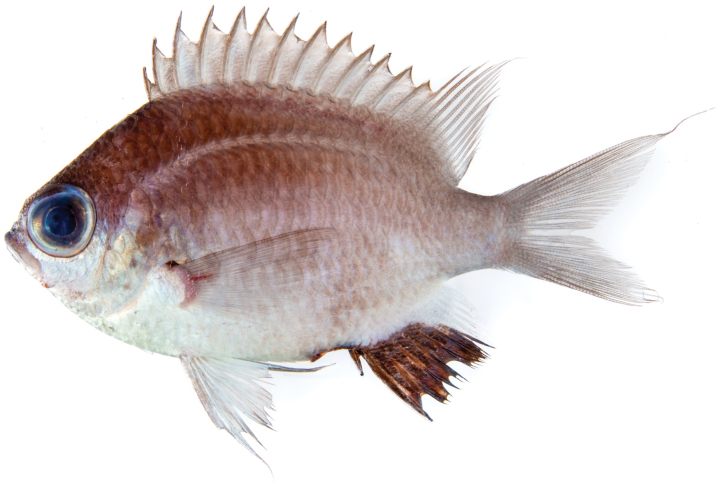
Holotype of *Chromisabadhah*, CAS 248401, 68.7 mm SL, shortly after collection. Photo by Luiz Rocha.

**Figure 2.**
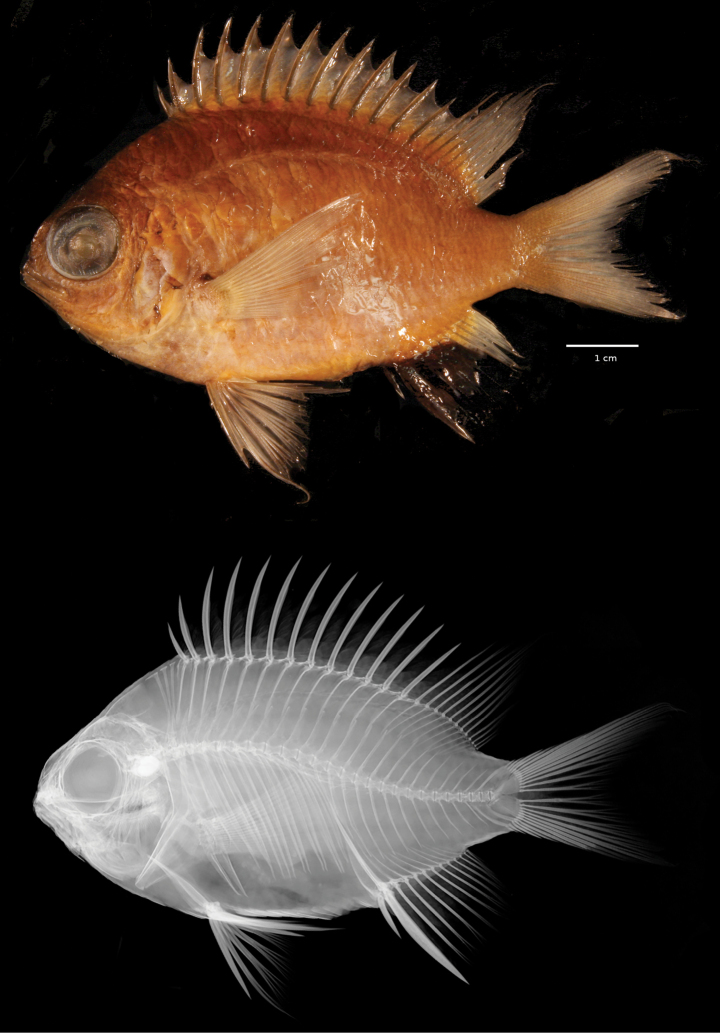
Preserved holotype and x-ray of *Chromisabadhah*, CAS 248401, 68.7 mm SL. Photo and x-ray by Jon Fong.

**Figure 3.**
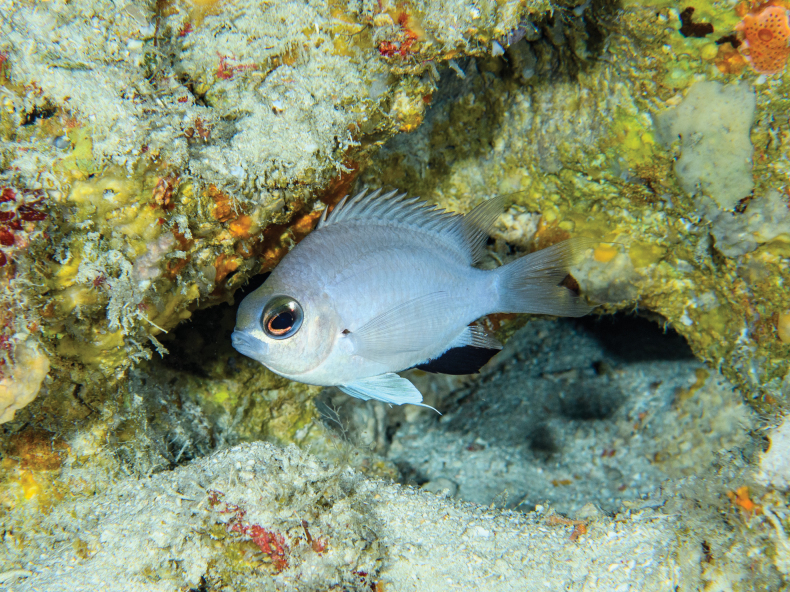
*Chromisabadhah* in its natural habitat in Faadhippolhu Atoll, Maldives, at approximately 110 m depth. Photo by Luiz Rocha.

Figs 1 , 2 , 3 , Table 1 Suggested Maldivian name: Abadhah Chromis Suggested English name: Perpetual Chromis

#### Type material.

***Holotype*** (Figs 1, 2) • CAS 248401 (field code LAR2969), 68.7 mm SL, GenBank PQ410417, Maafilaafushi, Faadhippolhu Atoll, 5°21'40"S, 73°24'45"E, hand nets 101 m, H.T. Pinheiro, B. Shepherd, L.A. Rocha, 15.XII.2022. ***Paratypes*** • CAS 248403 (field code LAR2968), 66.1 mm SL, GenBank PQ410416, same data as holotype • USNM 470765 (field code LAR2970), 64.5 mm SL, GenBank PQ410418, same data as holotype • CAS 248402 (field code LAR2965), 50.2 mm SL, Maafilaafushi, Faadhippolhu Atoll, 5°21'40"S, 73°24'45"E, hand nets 118 m, H.T. Pinheiro, B. Shepherd, L.A. Rocha, 14.XII.2022.

#### Diagnosis.

The following combination of characters distinguishes *Chromisabadhah* sp. nov. from all of its congeners: dorsal-fin rays XIII, 12–13; anal-fin rays II,11–12; pectoral-fin rays 17–18; tubed lateral-line scales 17; gill rakers 7+17–18 = 24–25; body pearly white; large black marking covering anterior two-thirds of anal fin; small black spot on upper edge of pectoral-fin base; no markings on caudal peduncle.

#### Description.

Dorsal-fin rays XIII, 12 (XIII, 12–13); anal-fin rays II, 11 (II, 12); all soft dorsal- and anal-fin rays branched, the last to base, the last two soft rays associated with a single complex pterygiophore; fourth (third) dorsal ray longest, 25.8% (20.8–28.2) SL; pectoral-fin rays 18|18 (17|18), the uppermost and lowermost unbranched; first pectoral ray the longest, 34.6% (31.6–34.5) SL; pelvic-fin rays I,5; principal caudal-fin rays 7+6 (6–7+6), the uppermost and lowermost unbranched; upper and lower procurrent caudal-fin rays 6, the anteriormost 3 rays (dorsally and ventrally) spiniform; tubed lateral-line scales 17; scales above lateral line to origin of dorsal fin 3; scales below lateral line to origin of anal fin 9; circumpeduncular scales 16 (16–18); gill rakers 7+17 = 24 (7+17–18 = 24–25); supraneural bones 3; vertebrae 11 precaudal + 15 caudal = 26.

Body moderately deep, depth 56.3% (55.0–59.0) SL, and compressed, the width 21.1% (18.3–20.2) SL; head length 35.6% (35.6–36.8) SL; profile of head slightly convex above orbit, nape slightly convex; snout length 9.6% (8.9–9.8) SL; eye large, orbit diameter 15.0% (12.6–14.2) SL; interorbital width 14.3% (12.2–13.4) SL; caudal-peduncle depth 14.3% (15.4–15.7) SL; caudal-peduncle length 8.0% (7.8–10.3) SL.

Mouth terminal, oblique, upper jaw angle about 45° to the horizontal axis of head and body; posterior edge of maxilla extending slightly beyond vertical at anterior edge of eye, upper jaw length 12.6% (10.5–11.5) in head length; teeth multi-serial, outer row of conical teeth in each jaw, much larger anteriorly; narrow band of villiform teeth lingual to outer row, in three irregular rows anteriorly, narrowing to a single row on side of jaws; tongue triangular with rounded tip; gill rakers long and slender, longest on lower limb near angle about half the length of gill filaments; anterior nostril relatively large with a short fleshy rim, more elevated on posterior edge and located at level of horizontal line through middle of pupil, slightly less than halfway between front of snout and anterior edge of orbit; posterior nostril much smaller and slit-shaped, located above and behind anterior nostril, close to edge of orbit. Opercle ending posteriorly in flat spine, the tip relatively acute and not obscured by scales; preopercular margin smooth, posterior margin extending dorsally to almost level of upper edge of orbit; suborbital with free lower margin extending nearly to a vertical at posterior edge of orbit.

Scales finely ctenoid; tubed portion of lateral line ending beneath rear portion of spinous dorsal fin (base of 13^th^ dorsal-fin spine); head scaled except lips; scaly sheath at base of dorsal and anal fins, progressively thicker towards body; column of scales on each membrane of dorsal fin, narrowing distally, those on spinous portion of dorsal fin progressively longer, reaching about two-thirds the distance to spine tips on posterior membranes; two or three columns of scales on anal-fin membranes, progressively smaller distally; small scales on caudal fin extending about one-third distance to posterior margin; small scales only on base of pectoral fins; median scaly process extending posteriorly from between bases of pelvic fins, its length about half that of pelvic spine; axillary scale above base of pelvic spine about half the length of pelvic spine. Origin of dorsal fin above second lateral-line scale; pre-dorsal length 41.9% (42.1–42.7) SL; spinous dorsal-fin base length 49.4% (46.2–50.9) SL; soft dorsal-fin base length 15.6% (14.5–16.3) SL; first dorsal spine 6.7% (8.5–10.3) SL; second dorsal spine 13.4% (13.2–15.9) SL; third dorsal spine 15.8% (16.8–18.4) SL; fourth dorsal spine 17.5% (17.7–21.4) SL; fifth dorsal spine 17.1% (17.7–21.0) SL; sixth dorsal spine 18.3% (17.6–20.5) SL; last dorsal spine 12.1% (11.8–14.4) SL; fourth dorsal ray longest, 25.8% (20.8–28.2) SL; first anal spine 7.5% (7.1–7.5) SL; second anal spine 23.5% (21.9–23.3) SL; longest anal-fin ray first, 22.2% (19.4–20.2); caudal fin forked, with filamentous extensions, its length 33.9% (32.1–39.2) SL, and concavity 15.0% (15.4–20.3) SL; first pectoral-fin ray longest, 34.6% (31.6–34.5) SL; pelvic spine 20.1% (20.6–23.2) SL; first pelvic soft ray 29.5% (29.5–32.6) SL.

***Color*.** In life (Fig. 3): Body pearly white with pale blue undertones, darker dorsally, with light gray pigment, especially between lateral line and dorsal fin. Belly and cheeks bright white. Row of scales below bottom half of eye very reflective. Bright silvery-blue circle surrounds darker, central part of iris. Area on head between eyes and above iris greenish silver. Lips pale blue. Anterior two-thirds of anal fin black. Distal portions of the soft dorsal, caudal, and posterior third of anal fin transparent. Small black spot on upper margin of pectoral-fin base. Pelvic fins pale blue to pearly white. Recently deceased and in alcohol (Figs 1, 2): Head and body overall gray in color (brown in alcohol), lighter ventrally. Dorsal, caudal, ventral, and pectoral fins pale gray (brown in alcohol) with distal portions translucent. Anal fin dark brown to black (black in alcohol); other black markings as described in life.

#### Habitat and distribution.

*Chromisabadhah* is only known from the Maldives. It has been recorded at eight locations spanning 180 km (from Faadhippolhu to Dhaalu Atoll) so we presume it should be widely distributed across the Maldivian Archipelago. The type specimens were collected on a steep slope between 101 and 118 m depth off Maafilaafushi Island, and other individuals were observed elsewhere between 80 and 120 m depth. Habitat complexity was medium to high (small crevices and caves) but of low relief, with an apparently high diversity of encrusting sponges.

#### Etymology.

The work that led to the discovery of this species was funded by the Rolex Perpetual Planet initiative through a Rolex Award for Enterprises to LAR. To honor this initiative, we name this species “abadhah” (pronounced aa-BAH-duh), which means “perpetual” in Dhivehi, the local language of the Maldives. We also hope that this species and its habitat remain perpetual. To be treated as a noun in apposition.

## ﻿Discussion

In addition to *Chromisabadhah*, three other species of *Chromis* have an overall pearly white body with black markings in life: *Chromisaxillaris* (Bennett, 1831), *Chromispelloura* Randall & Allen, 1982, and *Chromiswoodsi* Bruner & Arnam, 1979. All are mesophotic, having only been recorded below 30 m depth. *Chromisaxillaris* is the most widely distributed, being recorded in various locations between the western Indian Ocean and the western Pacific at depths of 60 to 100 m (Bruner and Arnam 1979; Allen 1991). *Chromispelloura* is found in the Red Sea at depths of 30 to 80 m, and is very similar in color and morphology to *C.axillaris* (Randall and Allen 1982). In addition to the black bar covering the caudal peduncle (also in the previous two species), the distal half of the anal fin of *C.woodsi* (found at 50 to 175 m depth in the coastal western Indian Ocean) also is solid black (Bruner and Arnam 1979). *Chromisabadhah* differs from all of these by lacking a black bar on the caudal peduncle, having 13 dorsal spines (all others have 14), and by a distribution apparently restricted to the Maldives.

Recently, Tang et al. (2021) revised the taxonomy of *Chromis* and reassigned several species to the genera *Azurina* and *Pycnochromis*. All mesophotic species remained in *Chromis* stricto sensu, and several authors have pointed out that most of them have 14 dorsal-fin spines (Pyle et al. 2008; Allen and Erdmann 2009; Tea et al. 2019). *Chromisabadhah* is unusual among mesophotic species by having 13; therefore, the number of dorsal-fin spines does not appear to diagnose a deep-reef clade, and a comprehensive molecular analysis that includes more species is needed to better understand the relationships among shallow and deep lineages.

## Supplementary Material

XML Treatment for Chromis abadhah

## ﻿Additional information

### Author ORCIDs

Luiz A. Rocha https://orcid.org/0000-0003-4011-569X

Hudson T. Pinheiro https://orcid.org/0000-0002-3143-1474

Claudia R. Rocha https://orcid.org/0000-0001-8069-6535

Bart Shepherd https://orcid.org/0000-0002-8918-1551

### Data availability

All of the data that support the findings of this study are available in the main text.
